# Turner’s syndrome mosaicism in girls with neurodevelopmental disorders: a cohort study and hypothesis

**DOI:** 10.1186/s13039-021-00529-2

**Published:** 2021-02-11

**Authors:** Svetlana G. Vorsanova, Alexey D. Kolotii, Oksana S. Kurinnaia, Victor S. Kravets, Irina A. Demidova, Ilya V. Soloviev, Yuri B. Yurov, Ivan Y. Iourov

**Affiliations:** 1 Veltischev Research and Clinical Institute for Pediatrics of the Pirogov Russian National Research Medical University, Ministry of Health of Russian Federation, Moscow, Russia 125412; 2 Yurov’s Laboratory of Molecular Genetics and Cytogenomics of the Brain, Mental Health Research Center, Moscow, Russia 115522; 3 Department of Medical Biological Disciplines, Belgorod State University, Belgorod, Russia 308015

**Keywords:** Aneuploidy, Chromosome X, Fluorescence in situ hybridization (FISH), Molecular cytogenetics, Phenotype, Somatic mosaicism, Turner’s syndrome

## Abstract

**Background:**

Turner’s syndrome is associated with either monosomy or a wide spectrum of structural rearrangements of chromosome X. Despite the interest in studying (somatic) chromosomal mosaicism, Turner’s syndrome mosaicism (TSM) remains to be fully described. This is especially true for the analysis of TSM in clinical cohorts (e.g. cohorts of individuals with neurodevelopmental disorders). Here, we present the results of studying TSM in a large cohort of girls with neurodevelopmental disorders and a hypothesis highlighting the diagnostic and prognostic value.

**Results:**

Turner’s syndrome-associated karyotypes were revealed in 111 (2.8%) of 4021 girls. Regular Turner’s syndrome-associated karyotypes were detected in 35 girls (0.9%). TSM was uncovered in 76 girls (1.9%). TSM manifested as mosaic aneuploidy (45,X/46,XX; 45,X/47,XXX/46,XX; 45,X/47,XXX) affected 47 girls (1.2%). Supernumerary marker chromosomes derived from chromosome X have been identified in 11 girls with TSM (0.3%). Isochromosomes iX(q) was found in 12 cases (0.3%); one case was non-mosaic. TSM associated with ring chromosomes was revealed in 5 girls (0.1%).

**Conclusion:**

The present cohort study provides data on the involvement of TSM in neurodevelopmental disorders among females. Thus, TSM may be an element of pathogenic cascades in brain diseases (i.e. neurodegenerative and psychiatric disorders). Our data allowed us to propose a hypothesis concerning ontogenetic variability of TSM levels. Accordingly, it appears that molecular cytogenetic monitoring of TSM, which is a likely risk factor/biomarker for adult-onset multifactorial diseases, is required.

## Background

Since the beginning of the last century, Turner’s syndrome has been systematically described in clinical and cytogenetic aspects [1–3]; the syndrome is occasionally designated as Shereshevsky-Turner syndrome in Russia and as Ullrich-Turner syndrome in Germany [1, 3]. This chromosomal disorder may result from monosomy of chromosome X (loss of whole chromosome X), mosaicism for X chromosome aneuploidy/loss and X chromosome aberrations, or structural rearrangements of X chromosome leading to a loss of syndrome-specific X chromosome loci [4, 5]. It is generally accepted that ~ 45% of Turner’s syndrome cases are associated with non-mosaic monosomy of chromosome X. The remainder is associated with mosaic aberrations of chromosome X (e.g. monosomy/disomy; monosomy/trisomy; monosomy/marker chromosome; monosomy/structural rearrangement(s); monosomy/isochromosome; monosomy/ring chromosome) in 20–35% of cases and with X chromosome rearrangements (isochromosome Xq, deletions of Xp and other exclusive rearrangements) in 10–35% of cases [5, 6]. Furthermore, mosaic X chromosome loss is repeatedly shown to be a possible element of pathogenic cascades in a variety of multifactorial diseases including brain (neurodevelopmental) disorders [7]. According to the available literature, there have been significant efforts for uncovering genotype–phenotype correlations in cohorts of females suffering from Turner’s syndrome with special attention to mosaic cases [6, 8–11]. However, Turner’s syndrome mosaicism (TSM) is usually ignored as a target in molecular (cyto)genetic analyses of neurodevelopmental (neurobehavioral) cohorts. Thus, TSM has been occasionally addressed in the context of neurodevelopmental disorders and molecular cytogenetic analysis of TSM in related clinical cohorts.

Here, we describe the study of TSM in a large cohort of girls with neurodevelopmental disorders and congenital anomalies by molecular cytogenetic techniques. Karyotypic and clinical data have been taken into account for understanding possible phenotypic outcomes of TSM. A hypothesis concerning ontogenetic instability of TSM suggesting diagnostic and prognostic significance of the analysis has been accordingly proposed.

## Materials and methods

### Patients

The cohort of girls with neurodevelopmental disorders (intellectual disability, autism and/or epilepsy) and congenital anomalies included 4021 individuals. The ages ranged between 4 months and 18 years. Molecular cytogenetic and molecular studies of the cohort were approved by the Ethics Committee of the Veltischev Research and Clinical Institute for Pediatrics of the Pirogov Russian National Research Medical University, Ministry of Health of Russian Federation, Moscow. Written informed consent was obtained from the parents of the patients.

### Cytogenetic analysis

Karyotyping by G- and C-banding was performed for all the girls from the cohort as detailed previously [12–14]. G-banding resolution was no less than 550 bands according to ISCN 2016 [15].

### FISH

Somatic chromosomal mosaicism was evaluated using fluorescence in situ hybridization (FISH) with chromosome-enumeration and site-specific DNA probes. X-chromosome-specific probe (DXZ1) was used in all the cases suggested to be affected by X chromosome monosomy (mosaic and non-mosaic). Site-specific DNA probes for the short arm and long arm of chromosome X (structural rearrangements) and chromosome-enumeration DNA probes for autosomes and chromosome Y (marker chromosomes and controlling during analysis of low-level mosaicism for rearrangements of chromosome X) were applied when needed. DNA probe labeling, in situ hybridization and detection was performed according to previously described protocols [16, 17]. Interphase FISH analysis was performed as repeatedly described in details [18–21]. Quantitative FISH was applied to metaphase plates and/or interphase nuclei for increasing the efficiency of scoring. The details of the analysis were previously described [19].

### SNP-array

SNP-array-analysis (molecular karyotyping) using CytoScan HD Arrays (Affymetrix, Santa Clara, CA, USA) consisting of about 2.7 million markers was performed as described earlier [22, 23]. Data were visualized using the Affymetrix ChAS (Chromosome Analysis Suite) software CytoScan® HD Array Version 4.1.0.90/r29400 (reference sequence—GRCh37/hg19).

## Results

**Fig. 1:**
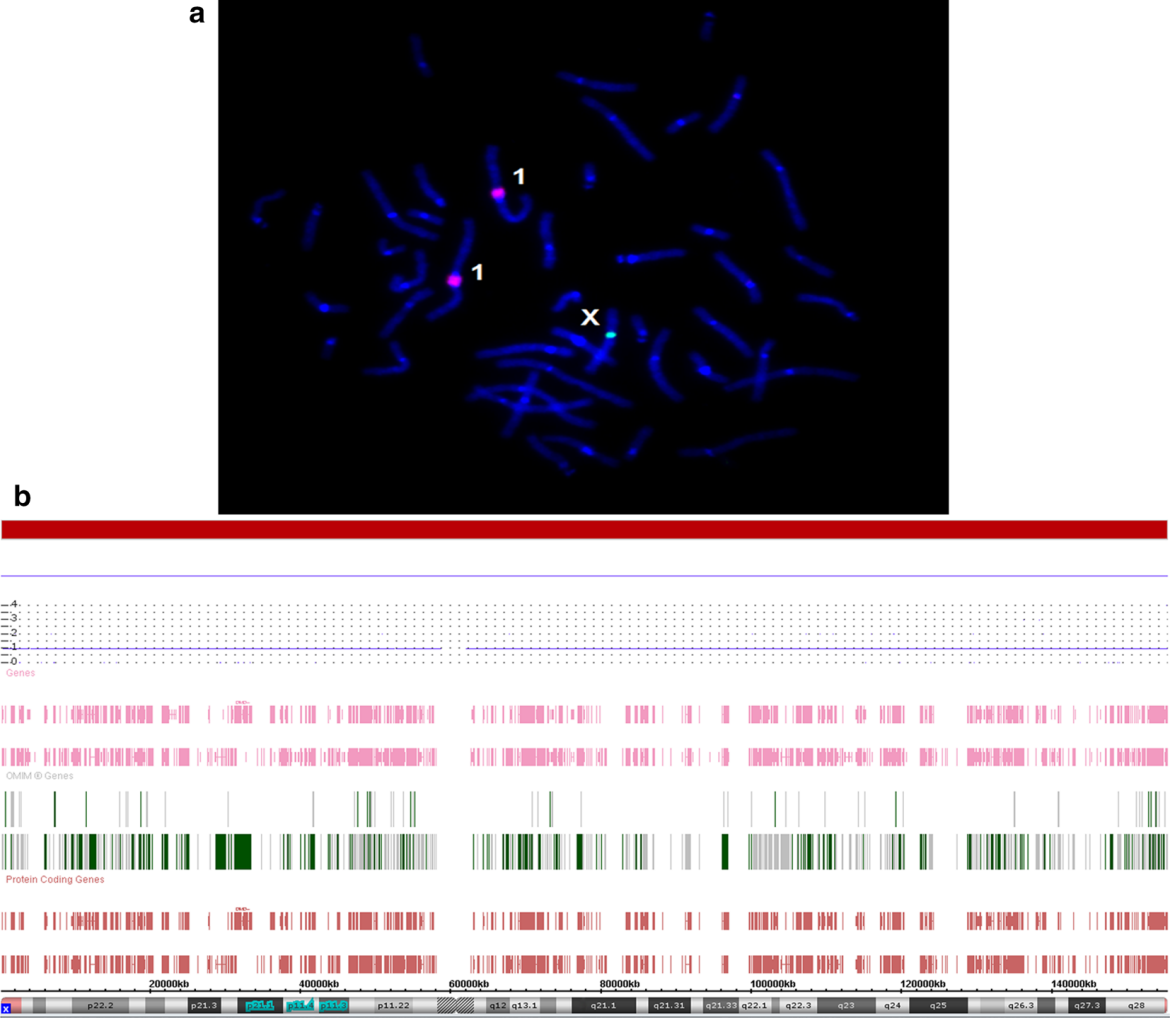
Molecular cytogenetic findings in a female with non-mosaic monosomy X; **a** FISH with a DXZ1 DNA probe (chromosome X, one green signal) and D1Z1 DNA probe (chromosomes 1, two red signals); **b** SNP-array results demonstrating non-mosaic X chromosome loss (regular monosomy X)

Cytogenetic and molecular cytogenetic analyses has identified Turner’s syndrome-associated karyotypes in 111 (2.8%) out of 4021 girls with neurodevelopmental disorders and congenital anomalies. Regular (non-mosaic) Turner’s syndrome-associated karyotypes have been detected in 35 girls (0.9% or 31.5% out of the whole group or girls with Turner’s syndrome-associated karyotypes, respectively). Twenty seven individuals (24.3%) have exhibited regular 45,X karyotypes (Fig. 1), whereas 8 patients (7.2%) have demonstrated structural rearrangements. TSM has been uncovered in 76 girls (1.9% or 68.5% out of the whole group or out of girls with Turner’s syndrome-associated karyotypes, respectively).

**Fig. 2:**
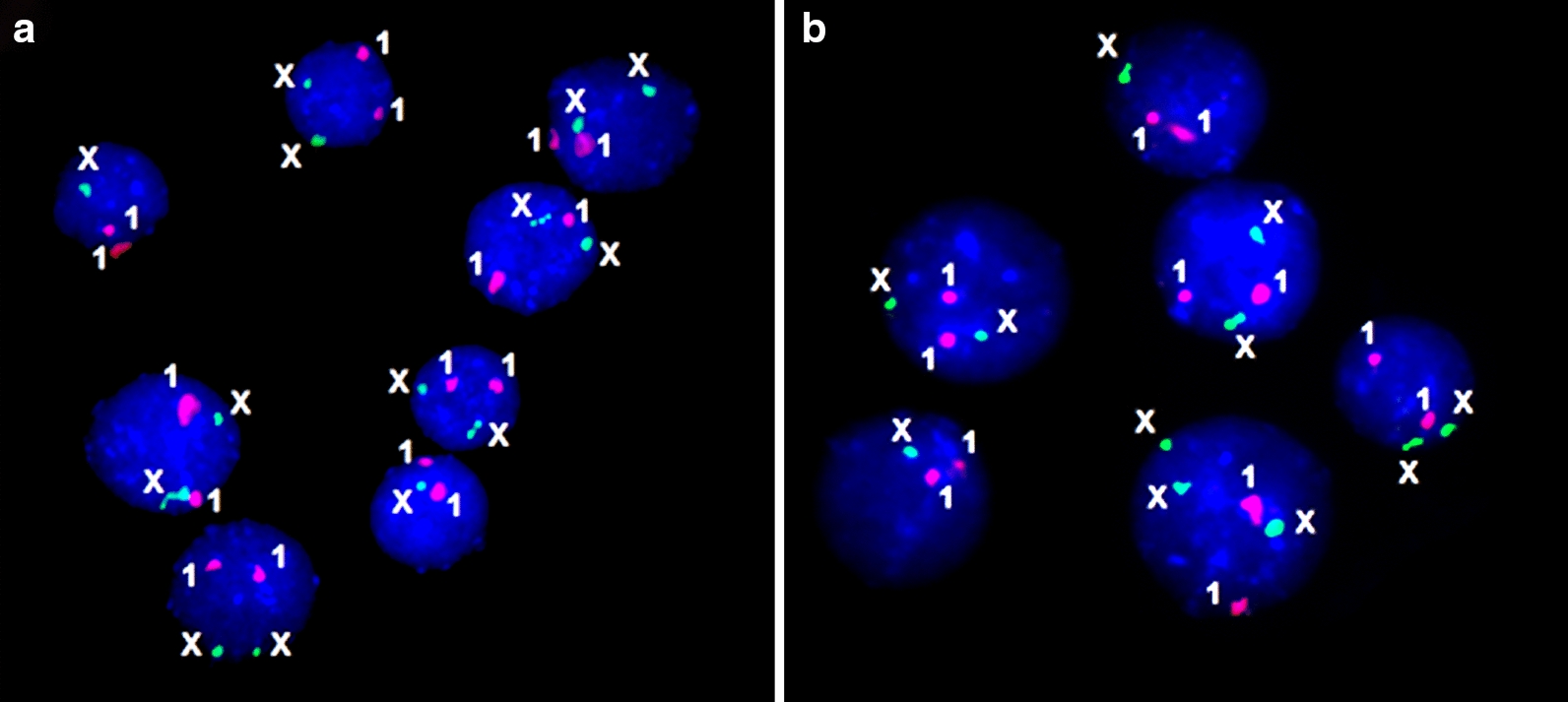
Interphase FISH with DXZ1 and D1Z1 DNA probes (chromosome X/green signals and chromosomes 1/red signals, respectively); **a** case of monosomy/disomy mosaicism; **b** case of monosomy/disomy/trisomy mosaicism

Interphase FISH (Fig. 2) has confirmed all the mosaic cases of TSM. TSM manifested as mosaic aneuploidy (i.e. 45,X/46,XX; 45,X/47,XXX/46,XX; or 45,X/47,XXX) has affected 47 girls (1.2%). Mosaicism levels varied from 5 to 90%. The remaining cases have been associated with mosaic marker chromosomes, isochromosomes and ring chromosomes.

Supernumerary marker chromosomes have been identified in 11 girls with TSM (0.3%). Table 1 provides an overview of TSM cases with marker chromosomes. It is noteworthy that all the marker chromosomes have derived from chromosome X as uncovered by FISH with X chromosome-specific probes. Autosome and Y-chromosome-specific probes have been also applied. Complex supernumerary marker chromosomes and marker chromosomes derived from chromosomes other than chromosome X have not been detected.

**Table 1:** Overview of TSM associated with supernumerary marker chromosomes

Chromosome abnormality	Cell proportions (%)	FISH results
45,X/46,X,+mar/46,XX	20/50/30	45,X.ishXp11.1q11.1(DXZ1×1)[10]/46,X,mar(X). ishXp11.1q11.1(DXZ1×2)[25] /46,XX.ishXp11.1q11.1(DXZ1×2[15]; nuc ishXcen(DXZ1×1)[243]/(DXZ1×2)[757]
45,X/46,X,+mar	38/62	45,X.ishXp11.1q11.1(DXZ1×1)[19]/46,X,mar(X). ishXp11.1q11.1(DXZ1×2)[31]; nuc ishXcen(DXZ1×1)[341]/(DXZ1×2)[659]
45,X/46,X,+ mar	54/46	45,X.ishXp11.1q11.1(DXZ1×1)[27]/46,X,mar(X). ishXp11.1q11.1(DXZ1×2)[23]; nuc ishXcen(DXZ1×1)[483]/(DXZ1×2)[517]
45,X/46,X,+mar	52/48	45,X.ishXp11.1q11.1(DXZ1×1)[26]/46,X,mar(X). ishXp11.1q11.1(DXZ1×2)[24]; nuc ishXcen(DXZ1×1)[448]/ (DXZ1×2)[552]
45,X/47,XX,+mar/46,XX	32/22/46	45,X.ishXp11.1q11.1(pYAM10-40×1)[16]/47,XX,mar(X). ishXp11.1q11.1(DXZ1×3)[11]/46,XX.ishXp11.1q11.1(DXZ1×2)[23]; nuc ishXcen(DXZ1×1)[389]/(DXZ1×3)[311]/(DXZ1×2)[300]
45,X/47,XXX/46,X,+mar*	38/42/20	47,XXX.ishXp11.1q11.1(DXZ1×3)[21]/45,X.ishXp11.1q11.1 (DXZ1×1)[19]/46,X,mar(X). ishXp11.1q11.1(pYAM1040×2)[10]; nuc ishXcen(DXZ1×3)[387]/(DXZ1×1)[303]/ (DXZ1×4)[110]/(DXZ1×2)[190]
45,X/46,X,+mar	72/28	45,X.ishXp11.1q11.1(DXZ1×1)[36]/46,X,mar(X).ish(DXZ1×2)[14]; nuc ishXcen(DXZ1×1)[387]/(DXZ1×2)[613]
45,X/46,X,+mar	52/48	46,X,mar(X).ishXp11.1q11.1(DXZ1×2)[26]/45,X.ishXp11.1q11.1 (DXZ1×1)[24]; nuc ishXcen(DXZ1×1)[477]/(DXZ1×2)[523]
45,X/46,X,+mar	38/62	45,X.ishXp11.1q11.1(DXZ1×1)[19]/46,X,mar(X). ishXp11.1q11.1(DXZ1×2)[31]; nuc ishXcen(DXZ1×1)[487]/(DXZ1×2)[513]
45,X/46,X,+mar	48/52	46,X,mar(X).ishXp11.1q11.1(DXZ1×2)[21]/45,X.ishXp11.1q11.1 (DXZ1×1)[19]; nuc ishXcen(DXZ1×1)[487]/(DXZ1×2)[513]
45,X/46,X,+mar/46,X,i(Xq)/47,XX,i(Xq)	30/26/24/20	45,X.ishXp11.1q11.1(DXZ1×1)[15]/46,X,mar(X).ishXp11.1q11.1(DXZ1×2)[13]/46,X,i(X)(q10)(DXZ1×2)[12]/47,XX,i(X)(q10) (DXZ1×3)[10]; nuc ishXcen(DXZ1×1)[277]/(DXZ1×3)[200]/ (DXZ1×2)[523]
45,X/46,X,mar	26/74	45,X.ishXp11.1q11.1(DXZ1×1)[13]/46,XX.ishXp11.1q11.1(DXZ1×2)[37]; nuc ishXcen(DXZ1×1)[209]/(DXZ1×2)[791]

^*^Cytogenetic analysis revealed cells with following karyotypes: 49,XXX,+mar,+mar; 48,XXX,+mar; 46,X,+mar; these karyotypes have not been detected by FISH

**Fig. 3:**
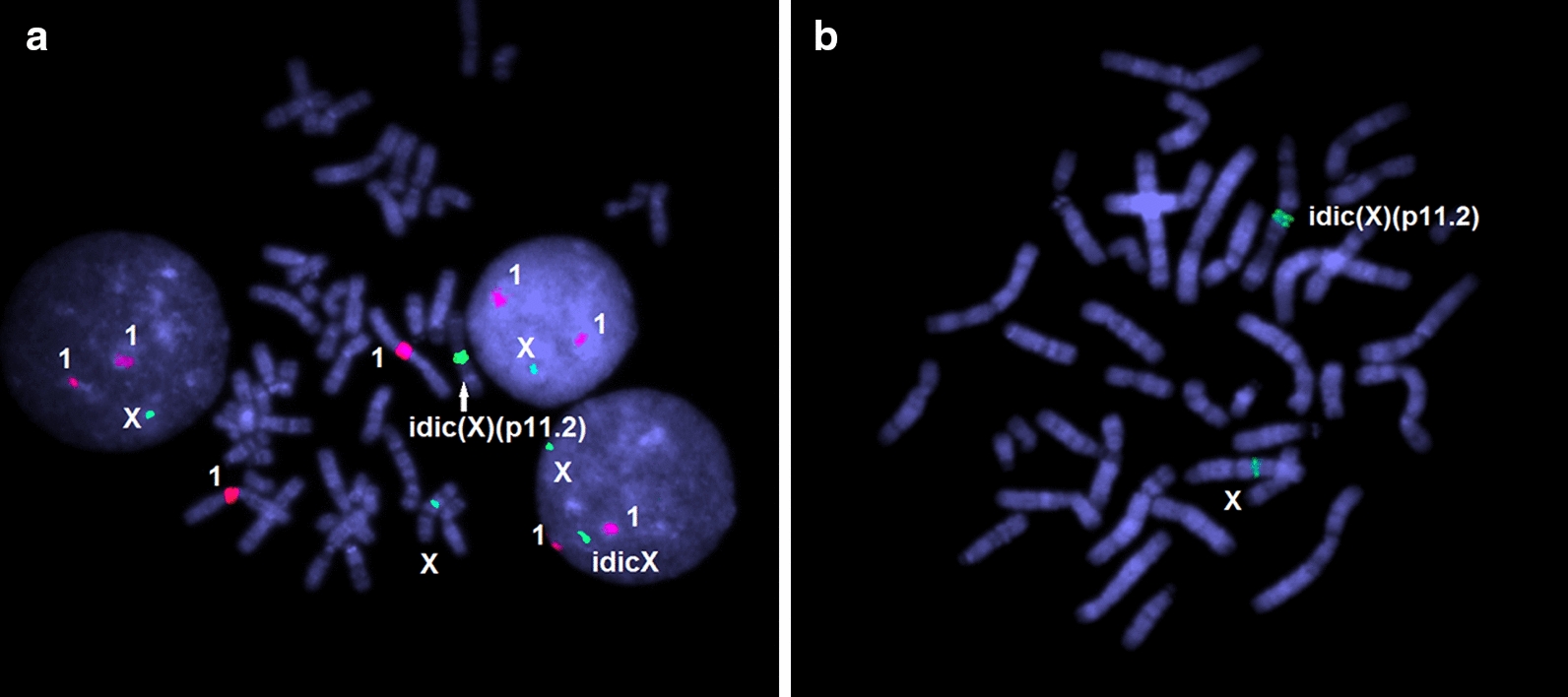
FISH on metaphase plates of a girl with an isochromosome X; **a** FISH with DXZ1 and D1Z1 DNA probes (chromosome X/green signals and chromosomes 1/red signals, respectively); note X chromosome loss in two interphase nuclei (left and upper right) indicating this case to be mosaic; **b** one-color FISH with DXZ1 DNA probe demonstrating isochromosome to be dicentric

Isochromosomes iX(q) (Fig. 3a) have been found in 12 cases (0.3%). One case has been non-mosaic. All the remaining cases (n = 11) have demonstrated mosaicism. In 3 cases of mosaicism for iX(q), FISH analysis has revealed that isochromosomes are dicentric (Fig. 3b). Table 2 gives an overview of isochromosomes X detected in the present cohort.

**Table 2:** Overview of TSM associated with isochromosomes

Chromosome abnormality	Cell proportions	FISH results
45,X/46,X,i(Xq)/46,XX	60/30/10	45,X.ishXp11.1q11.1(DXZ1×1)[30]/46,X,i(X).ishXp11.1q11.1(DXZ1×2)[15]/ 46,XX. ishXp11.1q11.1(DXZ1×2)[5]; nuc ishXcen(DXZ1×1)[321]/(DXZ1×2)[679]
45,X/46,X,i(Xq)/46,XX	40/26/34	45,X.ishXp11.1q11.1(DXZ1×1)[20] /46,X,i(X).ishXp11.1q11.1(DXZ1×2)[13]/46,XX.ishXp11.1q11.1(DXZ1×2)[17]; nuc ishXcen(DXZ1×1)[487]/(DXZ1×2)[513]
45,X/46,X,i(Xq)	32/68	46,X,i(X).ishXp11.1q11.1(DXZ1×2)[34]/45,X.ishXp11.1q11.1 (DXZ1×1)[16]; nuc ishXcen(DXZ1×1)[389]/(DXZ1×2)[211]
45,X/46,X,i(Xq)	74/26	45,X.ishXp11.1q11.1(DXZ1×1)[37]/46,X,i(X).ishXp11.1q11.1(DXZ1×2)[13]; nuc ishXcen(DXZ1×1)[717]/(DXZ1×2)[283]
45,X/46,X,i(Xq)	44/56	46,X,i(X).ishXp11.1q11.1(DXZ1×2)[28] /45,X.ishXp11.1q11.1(DXZ1×1)[22]; nuc ishXcen(DXZ1×1)[499]/(DXZ1×2)[501]
45,X/46,X,i(Xq)	36/64	46,X,i(X).ishXp11.1q11.1(DXZ1×2)[32]/45,X.ishXp11.1q11.1(DXZ1×1)[18]; nuc ishXcen(DXZ1×1)[214]/(DXZ1×2)[786]
45,X/46,X,idic(X)(q22.2)	24/76	46,X,idic(X).ishXp11.1q11.1(DXZ1×3)[38]/45,X.ishXp11.1q11.1(DXZ1×1)[12]; nuc ishXcen(DXZ1×3)[613]/(DXZ1×1)[387]
45,X/46,X,i(X)(q11.1)	43/57	46,X,i(X). ishXp11.1q11.1(DXZ1×2)[17]/45,X.ishXp11.1q11.1(DXZ1×1)[13]; nuc ishXcen(DXZ1×1)[413]/(DXZ1×2)[587]
45,X/46,X,idic(X)(p11.4)/ 46,XX	34/52/14	46,X,idic(X).ishXp11.1q11.1(DXZ1×3)[26]/45,X.ishXp11.1q11.1(DXZ1×1)[17]/46,XX. ishXp11.1q11.1(DXZ1×2)[7]; nuc ishXcen(DXZ1×1)[389]/(DXZ1×3)[300]/ (DXZ1×2)[211]
45,X/46,X,idic(X)(p11.3)	32/68	46,X,idic(X).ishXp11.1q11.1(DXZ1×3)[34]/45,X.ishXp11.1q11.1(DXZ1×1)[16]; nuc ishXcen(DXZ1×3)[623]/(DXZ1×1)[377]
45,X/46,X, + mar/46,X,i(Xq)/47,XX,i(Xq)	30/26/24/20	45,X.ishXp11.1q11.1(DXZ1×1)[15]/46,X,mar(X).ishXp11.1q11.1(DXZ1×2)[13]/46,X,i(X)(q10)(DXZ1×2)[12]/47,XX,i(X)(q10)(DXZ1×3)[10]; nuc ishXcen(DXZ1×1)[277]/(DXZ1×3)[200]/(DXZ1×2)[523]

**Fig. 4:**
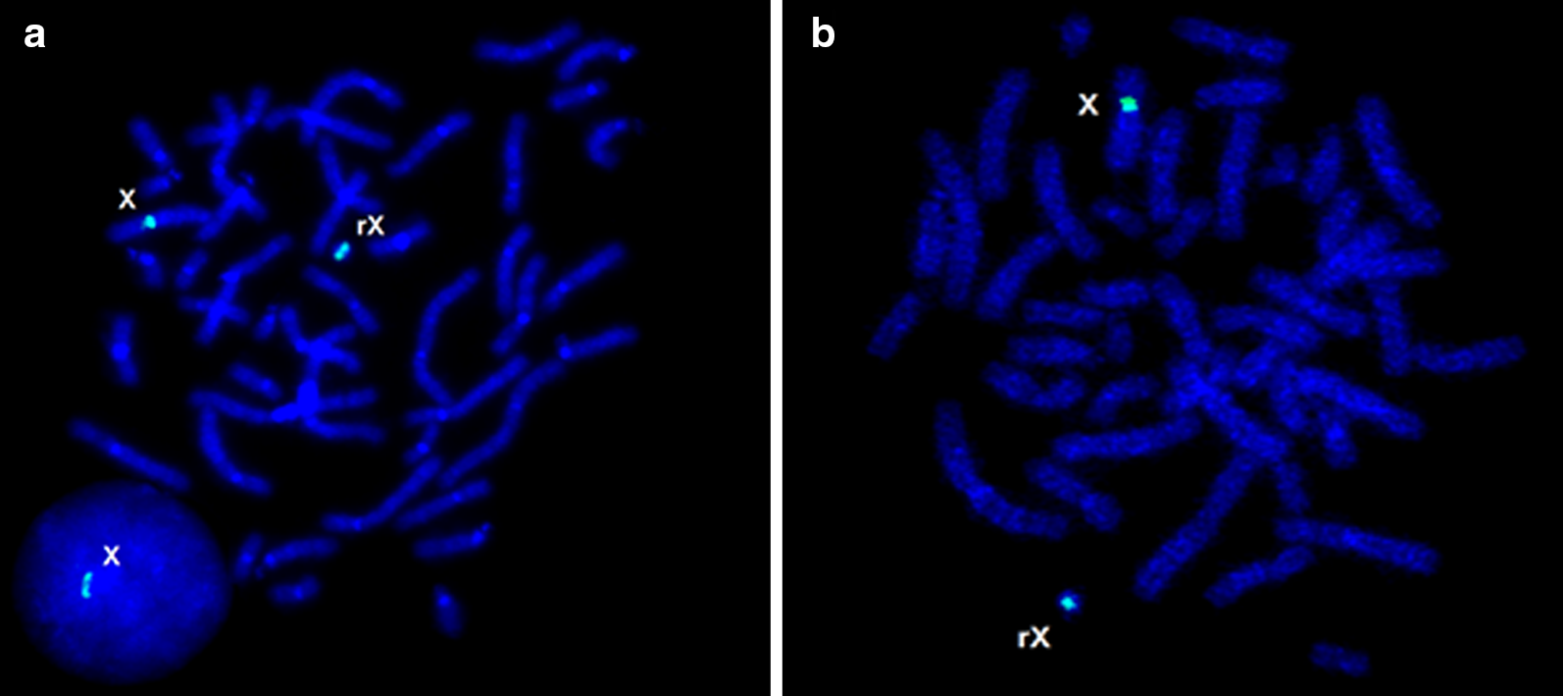
FISH with a DXZ1 DNA probe on metaphase plates of two girls with ring chromosome X (**a**, **b**); **a** note X chromosome loss in interphase nucleus indicating the case to be mosaic

Ring chromosomes (Fig. 4) have been revealed in 5 girls (0.1%). All cases have been associated with chromosomal mosaicism. Table 3 provides an overview of ring chromosomes X that have been found in girls with neurodevelopmental disorders and congenital anomalies from the present cohort.

**Table 3:** Overview of TSM associated with ring chromosomes

Chromosome abnormality	Cell proportions	FISH results
45,X/46,X,r(X)/46,XX	24/68/8	45,X.ishXp11.1q11.1(DXZ1×1)[12]/46,X,r(X).ishXp11.1q11.1(DXZ1×2)[34]/46,XX. ishXp11.1q11.1(DXZ1×2)[4]; nuc ishXcen(DXZ1×1)[209]/(DXZ1×2)[791]
45,X/46,X,r(X)(p21q21)	43/57	46,X,r(X).ishXp11.1q11.1(DXZ1×2)[23]/45,X.ishXp11.1q11.1(DXZ1×1)[17]; nuc ishXcen(DXZ1×1)[289]/(DXZ1×2)[211]
45,X/46,X,r(X)	28/72	46,X,r(X).ishXp11.1q11.1(DXZ1×2)[36]/45,X.ishXp11.1q11.1(DXZ1×1)[14]; nuc ishXcen(DXZ1×1)[389]/(DXZ1×2)[411]
45,X/46,X,r(X)	36/64	46,X,r(X).ishXp11.1q11.1(DXZ1×2)[32]/45,X.ishXp11.1q11.1(DXZ1×1)[18]; nuc ishXcen(DXZ1×1)[550]/(DXZ1×2)[450]
45,X/46,X,r(X)	42/58	46,X,r(X).ishXp11.1q11.1(DXZ1×2)[29]/45,X.ishXp11.1q11.1(DXZ1×1)[21]; nuc ishXcen(DXZ1×1)[489]/(DXZ1×2)[511]

Clinically, all 111 girls with Turner’s syndrome-associated karyotypes have demonstrated a range of neurodevelopmental phenotypes from minor neurobehavioral deficits to severe intellectual disability. Among other notable phenotypic features, we have observed short stature (n = 96; 86.5%), abnormal sexual development (n = 84; 75.7%), pterygium colli (n = 83; 74.8%), cardiac anomalies (n = 76; 68.5%) and renal abnormalities (n = 10; 9%). Karyotype-phenotype correlations (i.e. correlations between mosaicism rates and phenotypic outcomes) have not been established.

## Discussion

Turner’s syndrome represents a common chromosomal (gonosomal) syndrome (newborn prevalence: 5.9/1000) [24]. So far, this is the sole syndrome associated with non-mosaic monosomy in human [5, 6]. However, it is systematically hypothesized that liveborn children with non-mosaic 45,X karyotype are tissue-specific mosaics [6, 25, 26]. Recently, analyses of multiple tissues repeatedly supported this idea [27]. Since mosaicism is an important biomarker in Turner’s syndrome, high attention is paid to mosaic cases. Moreover, studies of TSM in clinical cohorts are a broad area of medical genetic research. This may be explained by the fact that gonosomal mosaicism is a phenomenon with global relevance to biomedicine [28]. Mostly, these studies are performed for cohorts of patients with reproductive problems or for children without specific clinical features [8, 29–31]. Surprisingly, despite of the presence of neurobehavioral and psychiatric endophenotypes in the clinical picture of Turner’s syndrome, analyses of TSM are exclusive in neurodevelopmental cohorts [32, 33]. The present study fills this gap providing a comprehensive analysis of TSM among females with neurodevelopmental disorders. Thus, this mosaicism type is involved in 1.9% of cases among neurodevelopmental disorders, i.e. such a phenotypically variable group of patients.

Somatic gonosomal mosaicism manifesting as aneuploidy is a contributor to the pathogenesis of numerous diseases [7, 34–37]. Here, we have shown that 1.2% of females with neurodevelopmental disorders are affected by mosaic X chromosome loss alone. Therefore, one can suggest that mosaicism for monosomy of chromosome X is a highly probable and relatively common mechanism of brain diseases in females. Supernumerary marker chromosomes derived from gonosomes have extremely variable phenotypic outcomes from asymptomatic carriage to irritant medical problems [38, 39]. Current report suggests that mosaicism for X chromosome loss and supernumerary marker chromosome X may be involved in pathogenesis of neurodevelopmental disorders in 0.3% of cases. TSM for X chromosome loss and isochromosomes X is suggested to have similar contribution to pathogenesis of neurodevelopmental disorders as TSM for supernumerary marker chromosomes X. TSM for X chromosome loss and ring chromosomes X is likely to contribute to pathogenesis of neurodevelopmental disorders in 0.1% of cases. The distribution of Turner’s syndrome-associated karyotypes among 111 girls is close to the results of the most comprehensive studies dedicated to the analysis of karyotypic heterogeneity in females with Turner’s syndrome [5, 6, 8, 10, 11]. However, according to the database of marker chromosomes managed by Prof. Thomas Liehr (http://cs-tl.de/DB/CA/sSMC/0-Start.html), 465/715 of Turner syndrome cases with the marker chromosome are derived from chromosome Y and only 246/715 cases are derived from chromosome X. The potential discrepancy between marker chromosome database and the data of our study may be related to the differences of cohorts addressed. Here, we address the cohort with relatively unspecific phenotypes (i.e. neurodevelopmental disorders and congenital anomalies), whereas marker chromosome database describes the distribution of derivative chromosomes among individuals with a specific phenotype (i.e. Turner’s syndrome). Alternatively, a more-or-less universal explanation referred to as the particularity of the cohort may be given. The lack of karyotype-phenotype correlations may be explained by unequal intertissular distribution of abnormal cells, which has been systematically reported previously [6–11, 26, 27].

It is import to note that somatic chromosome abnormalities (aneuploidy and structural rearrangements) are ontogenetically instable. In other words, the rates of mosaicism may increase with age mediating aging-related diseases and adverse aging effects [23, 40–44]. More importantly, X chromosome loss progresses during aging and is considered as a cytogenetic biomarker of aging [45–47]. Therefore, it is highly likely that the amount of cells affected by X chromosome loss will increase during the lifespan of girls with TSM. In the neurodevelopmental context, it is important to mention the involvement of mosaic X chromosome monosomy in neuropsychiatric diseases. Thus, X chromosome loss is associated with a variety of neurobehavioral diseases in children, adolescents and adults including familial cases [13, 14, 48, 49]. Schizophrenia and comorbid psychiatric disorders are commonly associated with X chromosome aneuploidy, which may specifically affect the brain [21, 50–52]. There are evidences for an involvement of aging-related X chromosome loss in the pathogenesis of Alzheimer’s disease [53–55]. In total, it is to conclude that X chromosome loss accumulated throughout ontogeny represents a mechanism for brain diseases with different ages of onset [25, 56]. Additionally, X chromosome loss has been shown to be associated with numerous diseases characterized by female preponderance (e.g. autoimmune diseases), which we have reviewed recently [7]. Therefore, unapparent phenotypic manifestations of TSM [57], should not be considered as a limitation for defining TSM as a biomarker for multifactorial diseases mediated by X chromosome aneuploidy. Furthermore, aging-related exhausting of molecular pathways guaranteeing chromosome stability and genetic-environmental interactions predispose to an increase in genome instability levels throughout ontogeny [23, 34, 58–62]. Moreover, chromosome abnormalities may initiate chromosome instability per se [23]. These observations allowed us to propose a hypothesis described below.

## Hypothesis

We hypothesize that levels of TSM are likely to increase in different tissues throughout the lifespan. Accordingly, this increase mediated by alterations to genome safeguarding pathways and genetic-environmental interactions would lead to occurrence of diseases associated with X chromosome loss. Among these diseases are neurobehavioral disorders, schizophrenia, dementia (e.g. Alzheimer’s disease) and autoimmune diseases. Taking into account social importance of these diseases, one may be aware about the application of TSM analysis for early (preclinical) diagnosis, prognosis and possible therapeutic interventions. In this context, we propose molecular cytogenetic monitoring of TSM for early detection of the increase of X chromosome loss levels throughout the life of the affected females. Additionally, system biology analyses of molecular and cellular pathways leading to the increase of X chromosome loss levels may offer a possibility to control/inhibit chromosomal mosaicism/instability. The combination of molecular cytogenetic monitoring and systems biology analysis of females with TSM would eventually lead to a possibility of successful evidence-based therapies of devastating multifactorial diseases.

## Conclusions

The incidence of Turner’s syndrome-associated karyotypes in girls with neurodevelopmental disorders achieves 2.8% (i.e. 20–30 in 1000 girls with intellectual disability, autism, epilepsy and/or congenital anomalies). It is to note that more than two-thirds of these girls exhibit TSM. Significant karyotypic heterogeneity mediated by TSM is observed in females with neurodevelopmental disorders. These data provide evidence for TSM contribution to the risk of brain diseases. Taking into account previous observations on somatic chromosomal mosaicism, we hypothesize that TSM proportions may ontogenetically change in favor of X chromosome loss. Thus, the occurrence of TSM might be a biomarker for adult-onset (multifactorial) diseases, which are mediated by X chromosome loss in an appreciable proportion of cases. Consequently, detecting and monitoring TSM is important for early diagnosis, prognosis and evidence-based therapeutic interventions in corresponding diseases.

## Data Availability

The data of this study are all included in the article.
